# Primary central nervous system CD20-negative diffuse large B-cell lymphoma: a case report

**DOI:** 10.1186/s12883-022-03031-3

**Published:** 2022-12-29

**Authors:** Shuai Luo, Xiang Huang, Yao Li, Jinjing Wang

**Affiliations:** Department of Pathology, Affiliated Hospital of Zunyi Medical University, Zunyi, Guizhou P.R. China

**Keywords:** CD20-negative, Diffuse large B-cell lymphoma, Central nervous system, Unclassifiable

## Abstract

**Background:**

CD20-negative diffuse large B-cell lymphoma is a very rare and heterogeneous invasive cancer characterized by chemical resistance and poor prognosis. Primary CD20-negative diffuse large B-cell lymphoma of the central nervous system is even rarer, presenting great challenges in pathological diagnosis and clinical treatment.

**Case presentation:**

We report a case of primary CD20-negative diffuse large B-cell lymphoma of the CNS in a 54-year-old woman admitted to the hospital with a headache lasting more than 10 days. CT and MRI scans showed right temporal lobe lymphoma. Microscopically, large infiltrating lymphoid cells that induced brain tissue damage were observed. Immunohistochemistry showed that the tumor cells were CD79a^+^, PAX-5^+^, MUM1^+^, and CD20^-^. The patient was diagnosed with lymphoma and transferred to an oncology hospital for chemotherapy. However, because the disease progressed rapidly, the patient died only after two rounds of chemotherapy.

**Conclusions:**

To the best of our knowledge, this is one of the first reported cases of unclassifiable CD20-negative diffuse large B-cell lymphoma located in the CNS. This case report aims to deepen the understanding of clinicopathological features of this type of lymphoma and expand the scope of this disease.

## Background

Primary central nervous system (CNS) lymphoma is a relatively rare, highly aggressive malignant tumor that accounts for approximately 1–2% of non-Hodgkin’s lymphoma. Of this percentage, diffuse large B-cell lymphoma (DLBCL) is the most common type of CNS lymphoma, accounting for about 84.6% of these cases [1, 2]. Approximately 98% of DLBCL cases express the B cell antigen marker, CD20 [3]. Of those patients, approximately, 76% will benefit from the rituximab + CHOP regimen. However, 1–2% of DLBCL cases do not express CD20 and are resistant to rituximab treatment. Furthermore, these cases are often accompanied by chemoresistance and extranodal organ invasion, resulting in a poor prognosis [4].

Here, we report the first case of CD20-negative DLBCL originating in the CNS. To improve the diagnostic accuracy of this rare disease, we comprehensively performed histopathological, immunophenotypic, and molecular characterizations. From these observations, an overview is given, and potential differential diagnoses are discussed.

## Case presentation

**Fig. 1:**
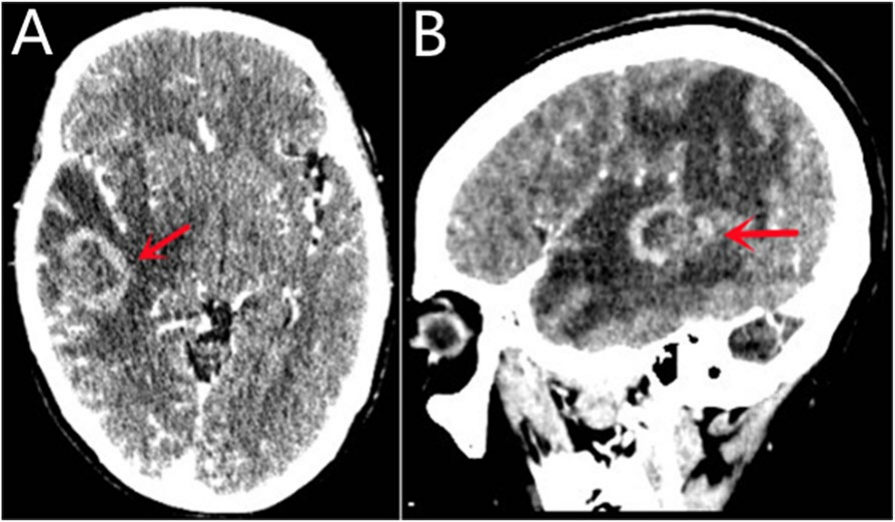
Brain MRI showed an irregular mass in the right temporal lobe, with obvious uneven enhancement on the enhanced scan, and a large area of non-enhancing edema around it, involving the basal ganglia in the (**A**) sagittal plane and (**B**) transverse plane

A 54-year-old female patient was admitted to the hospital due to a headache lasting for more than 10 days and did not have any indication of fever, night sweats, weight loss, or other B symptoms. Furthermore, the patient’s and family’s history was unremarkable. Physical examination of the patient revealed a clear consciousness, poor speech, and a GCS score of 15. Bilateral pupils were large and round with a diameter of approximately 3 mm. The patient’s indirect light reflex was sensitive and no bleeding was observed in the bilateral external auditory canal and nasal cavity. Spinal fluid flow and movement were not limited and no tenderness was noted. The patient’s limb muscle strength was assessed to be grade 5 and limb muscle tension was normal. The patient had a normal physiological reflex and the pathological reflex was negative. The IPI score was 3 (medium and high-risk group). Routine blood tests prior to admission revealed a white blood cell count of 4.7 × 10^9^/L, lymphocyte count of 1.09 × 10^9^/L, 0.23% lymphocytes, 165U/L lactate dehydrogenase (LDH), and 213 mg/L albumin. Serology revealed that the patient was HIV and HHV-8 negative. Cranial computed tomography (CT), as well as regular magnetic resonance imaging (MRI) in combination with contrast-enhanced MRI, showed an irregular mass measuring 31 × 27 × 20 mm in the right temporal lobe. The contrast-enhanced scan was unevenly enhanced, and a large area of non-enhanced edema involving the basal ganglia was observed. The right lateral ventricle and third ventricle were compressed, and the midline structure was shifted to the left by approximately 9.4 mm. CT and MRI findings suggested a high probability of right temporal lobe lymphoma (Fig. 1). Cranial functional magnetic resonance spectroscopy (MRS) indicated that the right temporal lobe tumor had increased Cho, a decreased Cr peak, a decreased NAA peak, and a maximum Ch/NAA ratio of 4.58, which suggests that the occupation of the right temporal lobe was malignant. The PET-CT data indicated that, characteristic of primary brain tumors, the right temporoparietal space-occupying lesions had increased metabolism (SUVmax 25) and large surrounding edema. No obvious areas of increased metabolism were found. The patient underwent a craniotomy to remove the intracranial, space-occupying lesion that was located on the deep surface of the right temporal lobe. The tumor was solid, translucent jelly-like, with clear borders, no capsule, minimal vasculature, and possessed a soft texture.

**Fig. 2:**
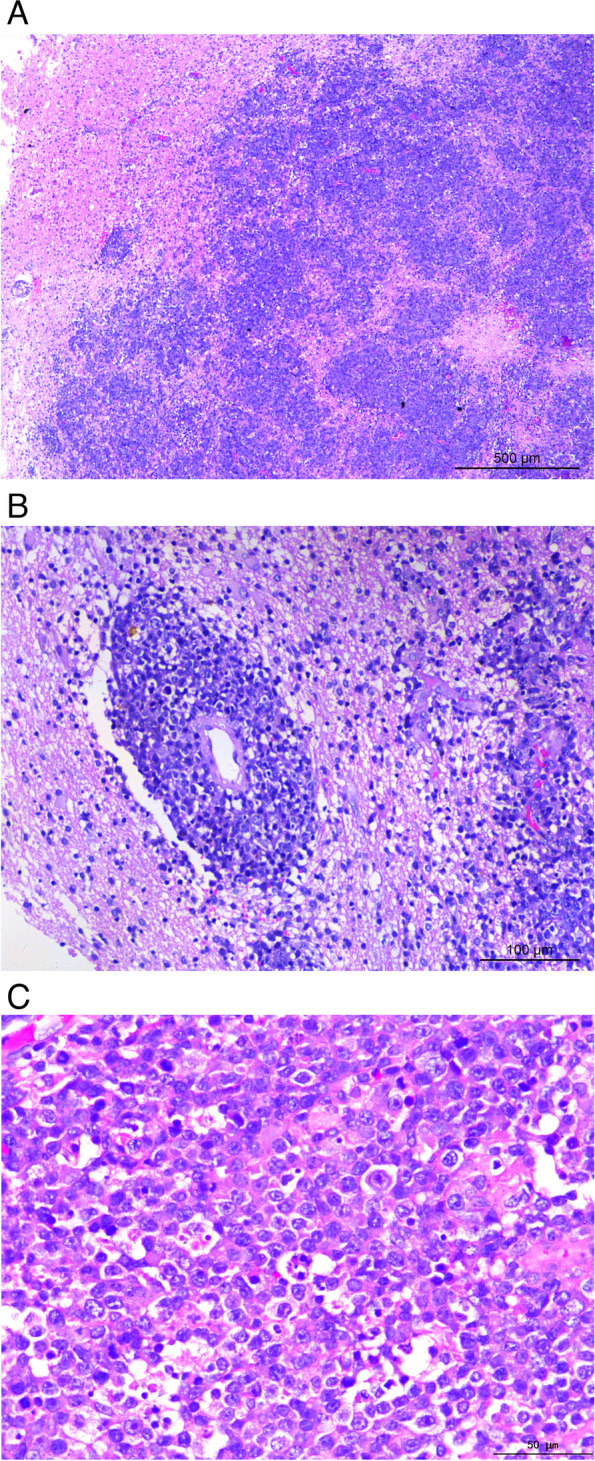
Histomorphology of the tumor indicated **A** diffuse infiltration of large lymphoid cells and destruction of brain tissue. No lymphoid follicular structure was observed. **B** Medium-to-large lymphoid cells are distributed around blood vessels in a ring-like arrangement. **C** Under the high magnification, the tumor cells were round or oval, with a clear nuclear membrane, vacuolated, close to 1 to 3 nucleoli near the nuclear membrane, and pathological mitotic figures can be seen

Gross pathological examination revealed an area of broken gray-white tissue (2.5 × 2 × 1 cm) with partial hyperemia, no capsule, and gray-white matter on the cut surface. Under low magnification, the brain tissue was diffusely infiltrated and severely damaged by many large lymphoid cells and no lymphoid follicle structures were observed (Fig. 2A). Additionally, local tumor interstitial microvascular proliferation, tumor cell arrangement around the blood vessels (Fig. 2B), and tumor cell infiltration of the blood vessel’s intracavity were noted. Under high magnification, the tumor cells were medium to large, round or oval, possessed clear nuclear membranes, displayed different nuclear staining characteristics, were variably vacuolated or possessing of irregular mass, exhibited nucleoli, and had 1 to 3 nucleoli with central or near nuclear membranes, cytoplasmic dichromatic or basophilic cells, centroblast-like and immunoblastic-like cells, and pathological mitotic cells are shown (Fig. 2C). From these morphological data, lymphoma was diagnosed, specifically favoring DLBCL.

**Fig. 3:**
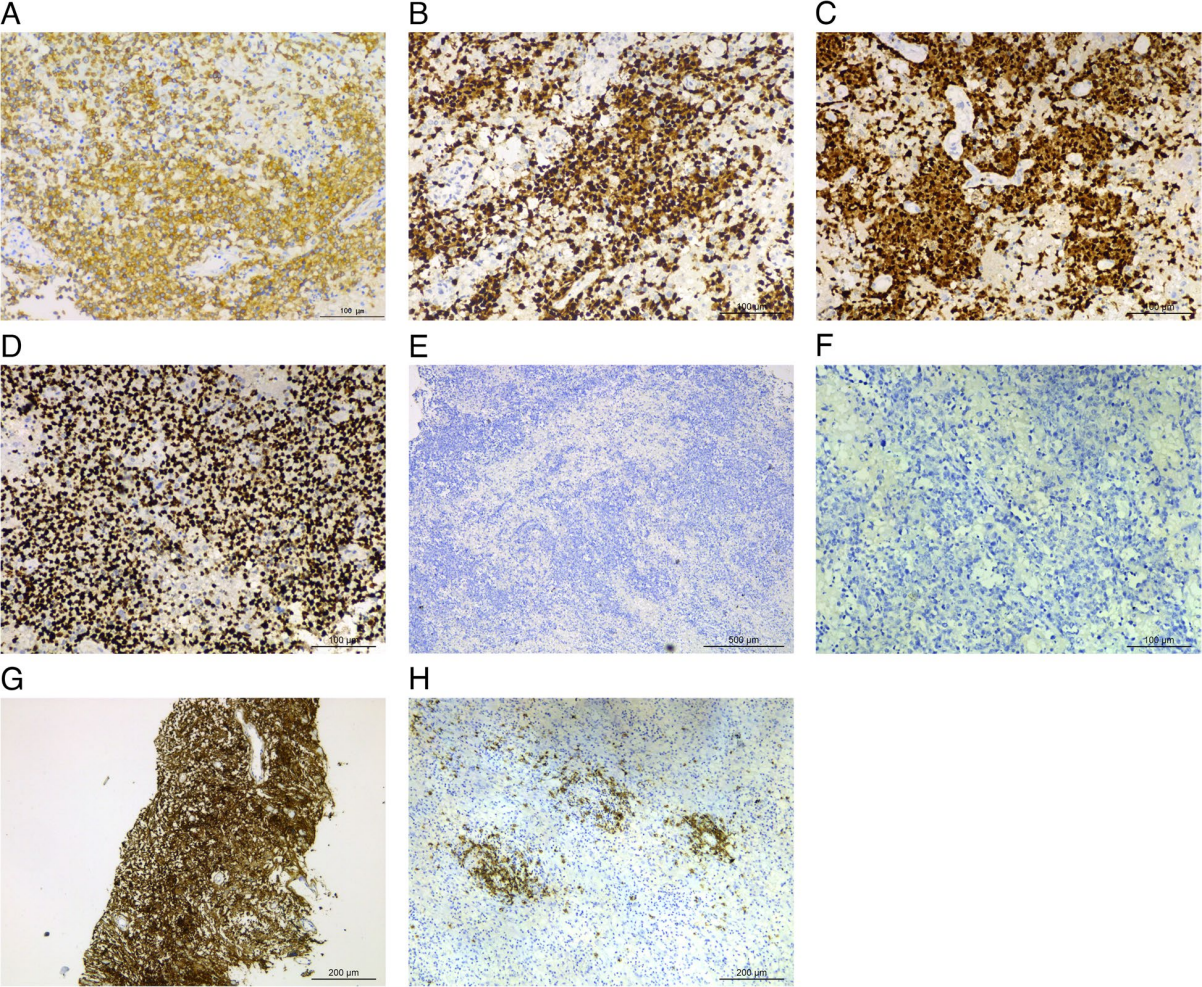
Immunohistochemical stains of large lymphoid cells. **A** CD79a (+); **B** PAX-5 (+); **C** MUM1 (+); **D** Ki-67 (90%+); **E** (×50) and **F** (×200) tumor tissue CD20 (-) ; **G** positive control CD20 (+); **H** CD20 (+) in reactive exuding lymphocytes in the brain tissue surrounding the tumor

Immunohistochemical assays identified the presence of large lymphoid cells by LCA^+++^, CD79a^+^ (Fig. 3A), PAX-5^+^ (Fig. 3B), MUM1^+^ (Fig. 3C), CD3^-^, and CD5^-^ staining that, together, are indicative of B cells involvement. Furthermore, the patient’s tissue was CD10^-^, Bcl-6^+^, and MUM1^+^, indicative that the cells did not originate from a germinal center. CyclinD1^-^, CD5^-^, SOX-11^-^, and Bcl-2^-^ staining data indicated that mantle cells and follicular cells likely did not contribute to the current phenotype. The EMA, GFAP, IDH1, P53, O1igo-2, S100, Syn, and CgA marks were all negative, thus excluding central nervous epithelial tumors such as astrocytoma and oligodendroglioma from consideration. Together, due to these data in combination with Ki-67 expression (90% positivity) (Fig. 3D), which is suggestive of active proliferation, the patient was diagnosed with DLBCL.

CD20 and CD21 staining of the tissues was negative. Initially, this result was considered to be a technical error that was introduced during the immunohistochemical analyses. However, upon repetition of the assay, and including a robust positive control, the results showed that the tissue remained CD20 negative (Fig. 3E-F) (the positive control was strongly positive (Fig. 3G)). Additionally, the reactive lymphocytes in the brain tissue surrounding the tumor were CD20 positive (Fig. 3H), which further excluded any false negatives that may have been present in the tissue-embedded wax block. Combined with morphological features and reliable immunohistochemical indicators, a definitive diagnosis of CD20-negative DLBCL was made.

To clarify the specific lymphoma type, additional immune indicators were assessed. The ALK, CD138, CD38, and EBER/ISH markers were all negative. Additionally, the Bcl-6 and EMA markers came back positive and negative, respectively. Laboratory serology revealed that the patient was HIV and HHV-8 negative. From these observations, the possibility that the current case was EBV-positive LBCL with plasmacytoid differentiation, primary effusion lymphoma (PEL), plasmablastoma (PBL), ALK^+^ LBCL, or HHV8^+^ DLBCL, NOS was dismissed. Combined with HE morphological features, these serological results are not consistent with anaplastic DLBCL. Additionally, the patient’s medical history was well recorded and no history of tumor and drug use (including rituximab) was present. Therefore, CD20-negative DLBCL with drug-induced CD20 antigen loss was excluded. Next-generation sequencing (NGS) was then used to detect 101 genes related to lymphoma. Of these genes, 11 gene mutations (Table 1), including *BTG2, ITPKB, TET2, FAT1, PIM1, CARD11, KMT2C, NOTCH1, KMT2D, SOCS1*, and *BCORL1* were noted.

**Table 1:** Gene mutations detected with NGS

Gene	Type	Allele Frequency(%)	Coding	Protein	Function	Transcript	Locus
BTG2	SNV	64.96	|c.19A>G	|p.Thr7Ala	|missense	NR_034151.1|NM_006763.2	chr1:203274753
ITPKB	SNV	70.99	c.964G>A	p.Ala322Thr	missense	NM_002221.3	chr1:226924196
TET2	SNV	42.44	c.3116C>T|	p.Ser1039Leu|	missense|	NM_001127208.2|NR_126420.1	chr4:106158215
FAT1	SNV	27.94	c.9514T>C	p.Phe3172Leu	missense	NM_005245.3	chr4:187532879
FAT1	SNV	25.8	c.6156A>C	p.Glu2052Asp	missense	NM_005245.3	chr4:187541584
PIM1	SNV	35.92	c.202C>T	p.His68Tyr	missense	NM_002648.3	chr6:37138769
CARD11	SNV	27.78	c.1010G>A	p.Arg337Gln	missense	NM_032415.5	chr7:2978320
CARD11	SNV	26.91	c.739A>G	p.Arg247Gly	missense	NM_032415.5	chr7:2979508
KMT2C	SNV	54.56	c.2512G>A	p.Gly838Ser	missense	NM_170606.2	chr7:151945007
NOTCH1	SNV	32.78	c.5476G>A	p.Glu1826Lys	missense	NM_017617.4	chr9:139396362
KMT2D	SNV	61.97	c.10045A>G	p.Met3349Val	missense	NM_003482.3	chr12:49431094
SOCS1	SNV	33.75	c.7G>C	p.Ala3Pro	missense	NM_003745.1	chr16:11349329
BCORL1	SNV	98.43	c.331T>C	p.Phe111Leu	missense	NM_021946.4	chrX:129147079

Together, these data in combination with the patient’s clinical history laboratory tests, HE morphological features, reliable immunohistochemical results, and genetic testing results, the diagnosis was CD20-negative DLBCL of the right temporal lobe. The specific type was noted as being unclassifiable/unclassified.

After the patient was diagnosed with lymphoma, he was transferred to the cancer hospital for chemotherapy. The patient was given high-dose methotrexate combined with temozolomide. The specific intravenous dosage of methotrexate (D1) was 4.5 g, and the specific oral dosage of temozolomide was 150 mg − 200 mg − 200 mg − 200 mg − 200 mg − 200 mg − 200 mg − 200 mg (D1-5). Urine PH was monitored during chemotherapy and was found to fall in the normal range. Additionally, the blood concentration of methotrexate was closely monitored. After 2 rounds of chemotherapy, the disease progressed rapidly and the patient died 4 months after the operation.

## Discussion and conclusions

Most CNS lymphomas are DLBCL. In addition to the histological features of general lymphomas, DLBCL often has notable characteristic histological features such as the presence of medium-to-large lymphoid cells that are distributed around blood vessels that can infiltrate arterioles in a ring-like arrangement. Additionally, spherical or cuff-shaped infiltration of small blood vessels can be observed. Previous studies have shown that this form of cellular infiltration can destroy blood vessels and allow cancerous cells to enter the lumen. In the current case, interstitial tumor cells were accompanied by significant microvascular proliferation and were seen to be distributed in a sleeve-like manner around the blood vessels.

CD20-negative DLBCL is a rare and heterogeneous group of aggressive lymphomas that have been further characterized into 5 sub-categories or types, namely: primary effusion lymphoma (PEL), plasmablastic lymphoma (PBL), ALK-positive large B-cell lymphoma, anaplastic lymphoma, lymphoma derived from human herpesvirus 8-related multicentric Castleman disease, and large B-cell lymphoma (HHV8^+^DLBCL, NOS) [5]. Additionally, plasmacytoid differentiated EBV-positive LBCL and CD20-negative DLBCL following rituximab treatment have also been reported [6]. These heterogeneous lymphomas all have immunoblasts as well as transcriptional profiles that are similar to that of plasma cells, and usually show high invasiveness, chemotherapy resistance, and low survival [5].

In addition to the above four types described above, in EBV-positive LBCL, unspecified (EBV^+^ DLBCL, NOS) often expresses MUM1, PAX-5, and CD20/CD79a. Furthermore, this sub-type does not express CD10, BCL-6, and CD15; and greater than 90% of cases may have EB virus infection. Lastly, these cases often exhibit CD20 deletion with marked plasmacytoid differentiation.

Previous studies reported that approximately 20% of CD20^+^ lymphomas were completely negative for CD20 after R-CHOP treatment, and that 6% were partially negative for CD20 [6, 7]. It is possible that Rituxan selectively attacks and destroys CD20^+^ tumor cells, resulting in the predominant proliferation of CD20^-^ tumor cells. Alternatively, the saturation of the concentration of Rituxan in serum may result in false negatives [8].

At the gene level, DLBCL cases are divided into two major molecular subtypes, germinal center B-cell-like (GCB) and activated B-cell-like (ABC). Additionally, there are a small number of unclassifiable sub-types according to specific, distinct gene expression profiles and cellular origins. With the development of gene sequencing technologies, more subtypes have been discreetly identified. The mechanism of *CD20* deletion is extremely complex. Studies have shown that *CD20* deletion in CD20-negative DLBCL is associated with gene changes and abnormal regulation, such as the deletion of the CD20-encoding gene, *MS4A1* [9], *FXPO1*, and other related transcription factors [10]. Furthermore, alterations to the ubiquitin-protease system have also been implicated to contribute to CD20-negative DLBCL [11]. In the current case, using next-generation sequencing, 11 gene mutations were identified. These included *BTG2, ITPKB, TET2, FAT1, PIM1, CARD11, KMT2C*, *NOTCH1*, *KMT2D*, *SOCS1*, and *BCORL1*. Consistent with the findings presented by Fukumura [12], hypermutation of the *BTG2* and *PIM1* genes in DLBCL of primary CNS was observed. Additionally, *NOTCH1* and *KMT2D* gene mutations were observed. These findings were consistent with the N1 typing data reported by Schmitz [13]. Based on classification data proposed by Lacy [14], the *TET2* and *SOCS1* gene mutations observed in the current study indicated that this DLBCL should be classified into an unclassified group other than the 5 described above. Enhanced mutations within the *CARD11* gene in the EZB-MYC-group were previously reported by George [15]. In agreement with mutations observed in tumors by Agata [16], mutations in the *BCORL1* and *TET2* genes were also observed. The gene and molecular heterogeneity of DLBCL are large, and few studies have investigated CD20-negative DLBCL-associated genetic mechanisms. Thus, the currently unclassifiable CD20-negative DLBCL disease pedigree and the mechanism of CD20 deletion need to be further expanded and confirmed.

In the current case, the above subtypes of DLBCL were excluded from the diagnosis based on clinical history, laboratory examination, histomorphological characteristics, and immunohistochemical results. The current case cannot be classified based on existing criteria, and as it occurred in the central nervous system, is extremely rare. Unclassifiable cases of CD20-negative DLBCL have been reported in foreign literature [17–19]; however, these unclassifiable cases have unique clinicopathological features. The current case is the first case of CNS, unclassifiable CD20-negative DLBCL.

In terms of treatment, the type of DLBCL has no target for rituximab due to CD20 deletion and is prone to chemotherapy resistance. Currently, a single CHOP regimen is used in clinical chemotherapy; however, the disease progresses rapidly and the prognosis is poor. Some scholars [20] have assessed the ESHAP regimen, high-dose liposomal doxorubicin + cyclophosphamide + vincristine + methylprednisolone chemotherapy, however, the effects were not satisfactory. The NCCN recommends that radiotherapy combined with second-line treatment such as bendamustine, lenalidomide, and other drugs can be tried, however, no reported benefit has been published to date. In the described case, the patient was treated with high-dose methotrexate plus temozolomide; however, the therapeutic effect was negligible and the disease was not well controlled. Taking the observations described in the current case report in combination with other available published treatment plans, it can be concluded that CD20-negative DLBCL is insensitive to a variety of chemotherapy drugs. The lack of sufficient knowledge of CD20-negative DLBCL pathogenesis as well as an effective treatment plan highlights the need for more in-depth mechanistic, clinical CD20-negative DLBCL studies that can be used to gain insights into this disease and improve patient outcomes.

In conclusion, CD20-negative DLBCL is rare and presents significant challenges in both diagnosis and treatment. In terms of diagnosis, clinical history, laboratory examinations, histomorphological characteristics, reliable immunohistochemical results, and more accurate immune marker examination techniques should be combined to robustly improve diagnosis. Additionally, the disease pedigree still needs to be further expanded. In terms of treatment, due to CD20 deletion, it is impossible to benefit from rituximab target therapy, and chemotherapy resistance is common. Novel treatment strategies that are based on a comprehensive understanding of the genetic mechanisms of unclassified DLBCLs are urgently required.

## Data Availability

All the data regarding the findings are available within the manuscript.

## Abbreviations

CT: Computed tomography
MRI: Magnetic resonance imaging
PET-CT: Positron Emission Tomography-Computed Tomography
NGS: Next-generation sequencing
NCCN: National Comprehensive Cancer Network
